# Efficacy of injectable toltrazuril-iron combination product and oral toltrazuril against early experimental infection of suckling piglets with *Cystoisospora suis*

**DOI:** 10.1186/s13071-019-3527-3

**Published:** 2019-05-28

**Authors:** Anja Joachim, Nicolas Guerra, Barbara Hinney, Adnan Hodžić, Hamadi Karembe, Aruna Shrestha, Daniel Sperling

**Affiliations:** 1 Department of Pathobiology, Institute of Parasitology, Vetmeduni Vienna, Veterinaerplatz 1, 1210 Wien, Austria; 2 Ceva Santé Animale, 10 avenue de la Ballastière, 33500 Libourne, France

**Keywords:** Pig, Swine, Coccidiosis, Forceris®, Baycox®, *Isospora suis*

## Abstract

**Background:**

Toltrazuril is frequently administered for the metaphylactic control of piglet cystoisosporosis. In a previous study, the efficacy of parenteral toltrazuril (45 mg/piglet, Group Forceris®) applied on the 2nd day of life (dol), and of oral toltrazuril (20 mg/kg of body weight, Group Baycox®) applied on the 4th dol was evaluated in an experimental model with *Cystoisospora suis* infection on the 3rd dol (late infection, LI). In a follow-up study, efficacy and safety were evaluated against infections with *C. suis* on the 1st dol (early infection, EI). Parameters included oocyst excretion and faecal consistency, body weight development, bacteriological examinations and animal health.

**Results:**

All control piglets (*n* = 12) shed oocysts and had diarrhoea, while parasite excretion was completely suppressed in both treatment groups (*n* = 13 each) and diarrhoea was reduced to a single animal (Forceris® group), resulting in significant differences for these parameters between the treated groups and the controls without significant differences among the treatment groups. No treatment-related adverse events were noted. Body weight gain was reduced in the control group during the acute phase of infection, resulting in significantly lower body weight on the 15th dol. Sows and piglets shed high numbers of *Escherichia coli*. *Clostridium perfringens* type A was only detected in low amounts in pooled litter samples. In comparison to the LI study oocyst shedding was more intense in the control animals in EI, while diarrhea was more frequent in LI. In both infection models a high efficacy of toltrazuril in the control of parasitological and clinical outcomes of experimental *C. suis* infection could be demonstrated. Since in the LI study high numbers of *Cl. perfringens* type A were detected, it is hypothesized that colonization with these opportunistic pathogens has synergistic effects with *C. suis* and may explain variable clinical outcomes in untreated animals as well as the sporadic occurrence of diarrhea in toltrazuril-treated piglets.

**Conclusions:**

Parenteral and oral toltrazuril administered on the 2nd or 4th dol is safe and effective against experimental infections with *C. suis* on the 1st to 3rd dol. The clinical outcome of experimental infections seems influenced by bacterial coinfections.

## Background

Toltrazuril is currently the only registered and effective option for the chemotherapeutic control of infections with *Cystoisospora suis* in suckling piglets in the European Union. Under experimental conditions, oral application on the 3rd to 5th day of life (dol) can efficiently control oocyst excretion, parasite induced diarrhea and the correlated depression in body weight gain (e.g. [1–4]). In a previous controlled experimental study, we compared oral application of toltrazuril on the 4th dol (combined with parenteral iron application on the 2nd dol for prevention of iron deficiency anemia) to parenteral treatment with a combinatorial product (Forceris®; toltrazuril + iron as gleptoferron) on the 2nd dol for the control of experimental *C. suis* infection on the 3rd dol [4]. Both applications were highly effective in controlling oocyst excretion (with complete suppression of oocyst excretion after application of the combination product), diarrhea and significantly improved weight gain.

*Cystoisospora suis* infections are induced by ingestion of sporulated oocysts from the environment, and they can take place at any age and in different management systems, including minimal disease or SPF herds [5–10]. Infection during the first days after birth is especially detrimental to piglets’ health [11–14], requiring early intervention [14]. We therefore conducted a follow-up study employing the same experimental model as before, except that infection with *C. suis* was carried out on the 1st dol. The aim of this study was to evaluate the efficacy of parental toltrazuril application on the 2nd dol and of oral application on the 4th dol against very early neonatal infections.

## Methods

A randomised, blinded controlled experimental study was conducted to evaluate the effect of different treatments with toltrazuril on the outcome of experimental *C. suis* infections in piglets on the first day of life. Methods and study design were essentially the same as before [4] except for the day of infection. A total of 38 piglets from three sows were enrolled in the study after initial health examination and randomly allocated to three groups by ascending birth weight and infected with 1000 sporulated oocysts of *C. suis* (strain Wien-I) each on the 1st dol. The Forceris® group (*n* = 13) received a fixed dose of 45 mg of toltrazuril + 200 mg of iron as gleptoferron (1.5 ml; Forceris®, Ceva Santé Animale, Libourne, France) applied intramuscularly (i.m.) on the 2nd dol. The Baycox® group (*n* = 13) received a fixed dose of 200 mg of iron as iron dextran (1 ml; Uniferon® 200, Virbac, Kolding, Denmark) i.m. on the 2nd dol and 20 mg/kg of body weight (BW) of toltrazuril (Baycox® 5%, Bayer Animal Health, Monheim, Germany) orally on the 4th dol according to the product label. The control group (*n* = 12) received only iron in the same manner as the Baycox® group. Piglets were observed for general health from the 1st to the 29th dol (end of study) and for post-treatment effects 1, 6 and 24 h post treatment. Individual faecal samples were obtained daily from the 5th to the 18th dol and body weight was determined after birth (1st dol) and weekly (8th, 15, 22nd and 29th dol) until the end of the study. Faecal samples were evaluated for consistency and scored [faecal score (FS): FS1, firm; FS 2, pasty; FS 3, semi-liquid; FS 4, liquid] and examined for the presence of oocysts by autofluorescence (AF). Positive samples were examined by a modified McMaster technique adapted to small amounts of samples to quantify the number of oocysts per gram of faeces (OpG) [15].

Results (body weight development, faecal consistency/diarrhoea, oocyst excretion) were compared by a Kruskal–Wallis test, and in cases of significant differences, groups were compared by Mann–Whitney U-test using GraphPad® Prism v.5.04 for Windows (GraphPad® Software, La Jolla, CA, USA). Significance was assumed for *P *< 0.05.

To evaluate the presence of other entero-pathogens in the litters, sows were sampled individually *ante partum* and examined for the presence of bacterial enteropathogens. On the first day of sampling (5th dol) a pooled faecal sample from each litter was examined for determination of bacterial causes of diarrhoea (*E. coli*, *Cl. perfringens*) or viral enteropathogens (porcine rotavirus A, porcine coronaviruses). In cases where animals of the treated groups (Forceris® or Baycox®) showed diarrhoea after treatment, individual samples (first day of diarrhoea) from the affected piglet and an age-matched healthy animal from the same group were sent for bacteriological examination including specification of *E. coli* virulence factors and *Cl. perfringens* type A - beta 2 toxin.

## Results

Oocyst excretion was observed by autofluorescence (AF) and McMaster in all piglets of the control group. AF detected that oocyst shedding lasted 7.1 days on average; McMaster countable oocysts were observed for 6.1 days on average (Table 1). The prevalence of McMaster countable excretion in the control group reached a first peak on the 7th day post-infection (dpi) and a second one on the 12th dpi. The maximum oocyst shedding in the control group was 232,434 oocysts per gram of faeces (OpG) on the 8th dpi. None of the piglets from the Forceris® or the Baycox® groups shed oocysts detectable by AF or McMaster (Table 1). Consequently, the two treatment groups, Forceris® and Baycox®, were significantly different from the control group but not between each other regarding oocyst excretion (Table 2).

**Table 1:** Comparison of groups: Oocyst excretion (autofluorescence, McMaster, oocysts per gram of faeces), diarrhoea (faecal scores 3 and 4) and body weight gain

	Forceris®	Baycox®	Control
No. of piglets	13	13	11
No. of sampling days	179	180	154
Oocyst excretion			
No. of piglets positive in AF/MM (%)	0 (0.0)	0 (0)	11 (100)
Mean excretion days/piglet AF (min-max)	0	0	7.1 (3–12)
Mean excretion days/piglet MM (min-max)	0	0	6.1 (2–11)
No. of excretion days AF (%)	0 (0)	0 (0)	79 (51.3)
No. of excretion days MM (%)	0 (0)	0 (0)	67 (43.5)
Mean area under the curve for OpG	0	0	17,162
Faecal consistency			
No. of piglets with diarrhoea (%)	1 (7.7)	0 (0)	11 (100)
Mean diarrhoea days/piglet (min-max)	0.4 (0–5)	0	3.6 (1–6)
No. of diarrhoea days (%)	5 (2.8)	0 (0.0)	40 (26.0)
Mean area under the curve for FS	16.0	14.4	26.7
Body weight development			
Mean BWG (g) 1st-29th dol (%)	5701.7^a^ (501.8)	5484.6 (497.1)	4894.5 (439.8)
Mean daily BWG (g) 1st-29th dol	203.6	195.9	174.8
Mean daily BWG (g) 8th-15th dol	212.3	206.7	63.9

^a^For one piglet, no values for BW22 and BW29 were available

*Abbreviations*: AF, autofluorescence; MM, McMaster; OpG, oocysts per gram of faeces; FS, faecal score; BWG, body weight gain; dol, day of life

**Table 2:** Statistical evaluation: *P*-values are given for different parameters (Kruskal–Wallis test and Mann–Whitney U-test when *P *< α; α = 0.05). Degrees of freedom *df* = 2 for all parameters

Parameter	Forceris® *vs* control	Baycox® *vs* control	Forceris® *vs* Baycox®	*χ* ^2^
Oocyst excretion				
Number of days with AF detectable excretion	< 0.0001	< 0.0001	> 0.9999	34.6
Number of days with MM countable excretion	< 0.0001	< 0.0001	> 0.9999	34.6
AF detectable excretion present or not	< 0.0001	< 0.0001	> 0.9999	36.0
MM countable excretion present or not	< 0.0001	< 0.0001	> 0.9999	36.0
Area under the curve for oocysts per gram of faeces	< 0.0001	< 0.0001	> 0.9999	185.7
Faecal consistency				
Area under the curve for FS	< 0.0001	< 0.0001	0.3810	20.7
Number of days with diarrhoea	< 0.0001	< 0.0001	> 0.9999	29.3
Diarrhoea present or not	< 0.0001	< 0.0001	> 0.9999	31.9
Body weight development				
Body weights 1st dol	Kruskal–Wallis test: *α* = 0.8795			0.257
Body weights 8th dol	Kruskal–Wallis test: *α* = 0.1554			3.723
Body weights 15th dol	0.0069	0.0041	0.9703	9.659
Body weights 22nd dol	Kruskal–Wallis test: *α* = 0.1440			4.518
Body weights 29th dol	Kruskal–Wallis test: *α* = 0.3730			1.972
Daily body weight gain 8th–29th dol	Kruskal–Wallis test: *α* = 0.2891			2.482
Daily body weight gain 8th–15th dol	< 0.0001	< 0.0001	0.9999	18.56

The average faecal score (FS) increased above 2 in the control group from 8 to 13 dpi with a peak of 3.1 at 9 dpi, while in the treated groups the mean FS never rose above 2. The maximum prevalence of diarrhoea was 72.7% in the control group (9 and 10 dpi) with an average duration of 3.6 days, while in the Forceris® group only a single animal showed diarrhoea for 5 days (Table 1). Faecal score 4 (watery diarrhoea) was observed in 72.7% of the control animals (average duration: 1.5 days) while in the Forceris® group the single animal with diarrhoea had FS 4 for two days. In the Baycox® group FS 3 and 4 were not observed (Table 1). The area under the curve for FS, the number of days with diarrhoea and the number of piglets with diarrhoea were significantly reduced in the Forceris® and Baycox® groups compared to the control group without significant differences between the treatment groups (Table 2).

**Fig. 1:**
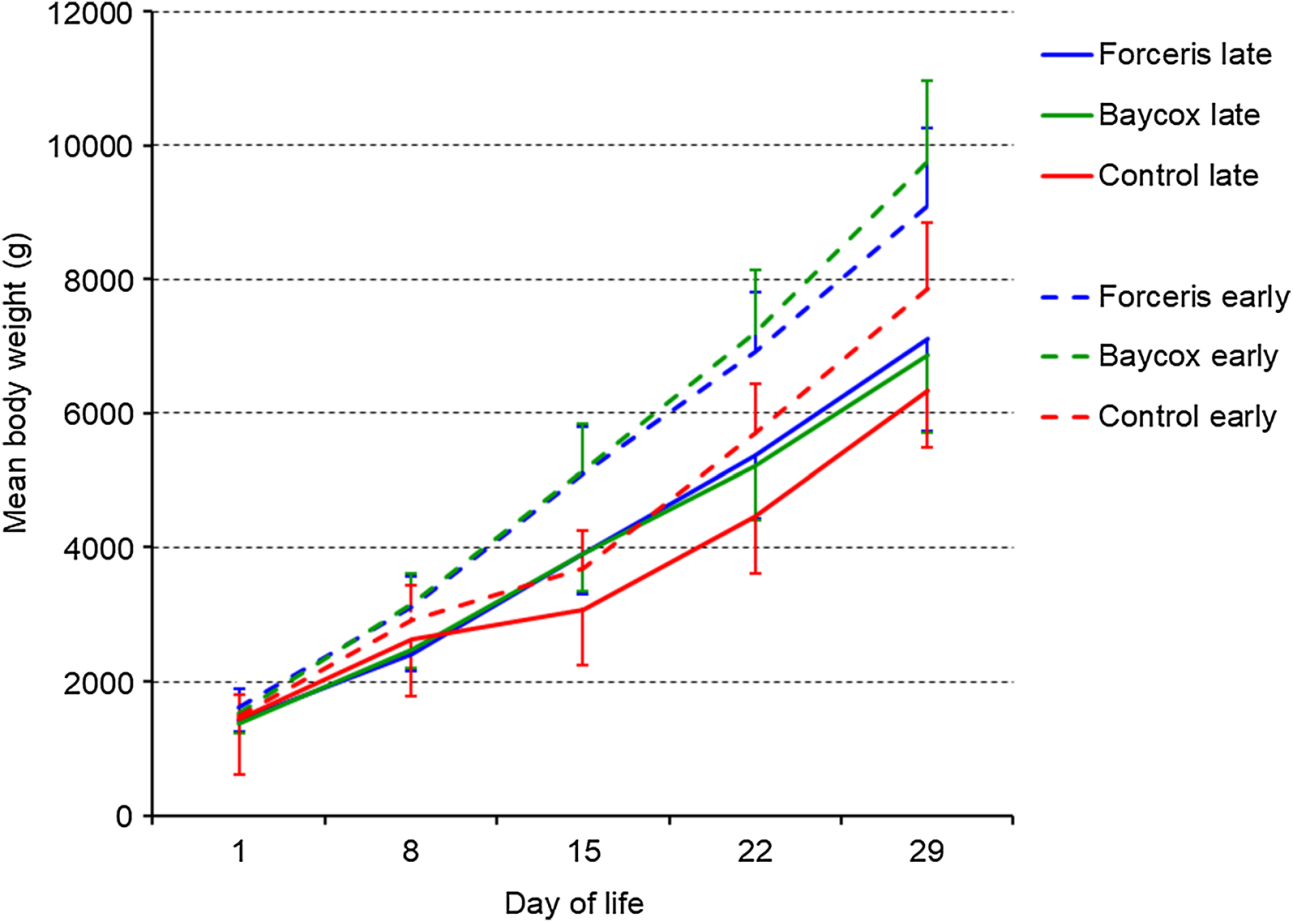
Body weight development during the study (with error bars). Dotted lines represent the groups in the previous trial [4]

Body weights were not significantly different between the groups on SD 1, the day of randomisation [Kruskal–Wallis test (α = 0.05); *χ*^2^ = 0.2569, *df* = 2, *P* = 0.8795]. Daily body weight gain and total weight gain from the 1st to 29th dol were reduced in the control group due to a severe depression of weight gain in the acute phase of infection (8th to 15th dol) when the control group only increased by 447.3 g on average compared to 1446.9 g in the Baycox® group and 1486.2 g in the Forceris® group (Table 1, Fig. 1). The control group had significantly lower body weights on the 15th dol compared to the two treatment groups without significant differences between the two treatment groups. This effect was even more noticeable for the daily body weight gain from the 8th to 15th dol (Tables 1, 2).

All sows sampled 5–10 days before farrowing shed high numbers of *Escherichia coli* (virulence factors detected: fimH, PapC, iucD and cnf1 in all sows, astA in sow no. 1) but no *Clostridium perfringens*. Piglets aged five days excreted high numbers of *E. coli* (virulence factors detected: fimH in litters 1 and 2, iucD in all 3 litters) and low numbers of *Cl. perfringens* type A (β2-toxin positive) on the 5th dol as evaluated by litter. The piglet from the Forceris® group that had diarrhoea for 5 days excreted a low number of *E. coli* (fimH, iucD) on the 6th dol and no *Cl. perfringens*. The healthy and age matched littermate shed low numbers of *E. coli* positive for fimH and no *Cl. perfringens*. No viral infections were detected.

One piglet from the control group was euthanized on SD 5 due to severe lameness after an accident and was excluded from further analysis. One piglet from the Forceris® group was removed due to injury on SD 19 but all data available were included. No animal showed treatment-related reactions that required veterinary intervention. No swelling or other reactions to the injections were observed.

**Fig. 2:**
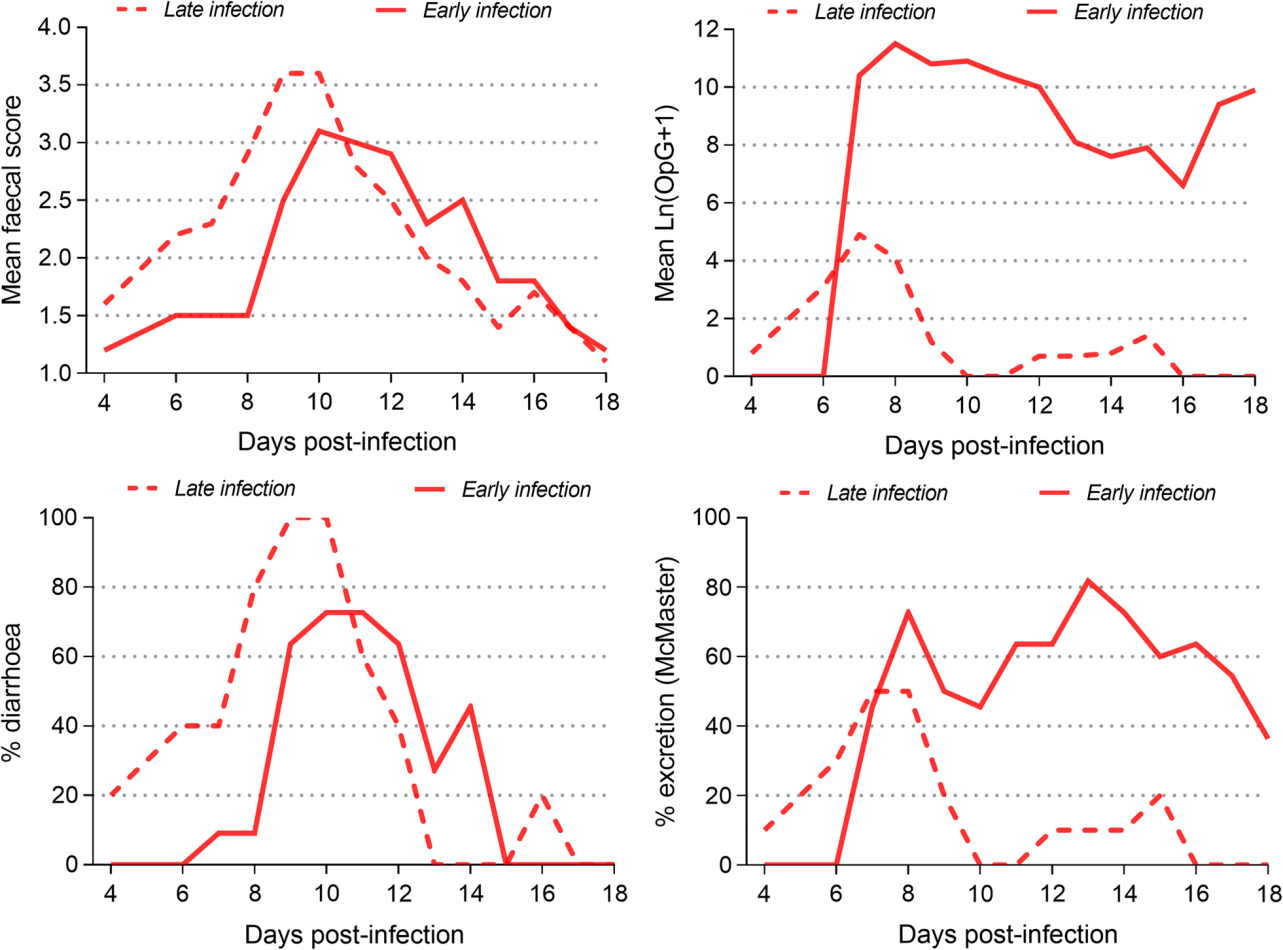
Comparison of faecal consistency and oocyst excretion between early (this trial) and late [4] infections (only controls)

In a previous experimental trial [4] the same study design was evaluated except that infection with *C. suis* oocysts was carried out on the 3rd instead of the 1st dol. Although the data cannot be analysed statistically since litters differed, the results are comparable due to the identical study design (Table 3; Figs. 1, 2). Oocyst excretion was distinctly more intense in the control group after infection on the 1st dol. The body weight development was generally much slower in the present trial (Fig. 1), indicating a strong litter effect on this parameter. Despite increased parasite excretion, diarrhoea was less intense after early infection. Regarding bacteriological results, *E. coli* and *Cl. perfringens* Type A (beta 2 toxin-positive) were present in both trials but much more abundant in the trial employing the later infection day, and more *E. coli* virulence factors were detected in that trial (Table 4).

**Table 3:** Comparison of the results of trials with the same design but different infection days for parasitological and clinical parameters

Parameters	Infection on 1st dol (this study)	Infection on 3rd dol [4]
Oocyst excretion (control groups)		
% of samples positive for oocysts (MM)	43.5	15.0
% of samples positive for oocysts (AF)	51.3	22.0
Mean duration of oocyst excretion (MM)	6.1	4.0
Mean duration of oocyst excretion (AF)	7.1	3.5
Maximum OpG (dol)	232,434 (9)	49,000 (9)
No. of McMaster-positive samples with OpG > 10,000 / all MM positive samples (%)	25/67 (37.3)	10/26 (38.5)
Faecal consistency (control groups)		
% of samples with diarrhoea	26.0	35.7
% of samples with FS 4	72.7	70.0
Mean diarrhoea days	3.6	5.0
Mean days with FS 4	1.5	3.0

*Abbreviations*: AF, autofluorescence; MM, McMaster; FS, faecal score; dol, day of life

**Table 4:** Comparison of the results of trials with the same design but different infection days for bacteriological results

Bacteriology (bacterial load and virulence factors)				
	*E. coli* (virulence factors)		*Cl. perfringens* (type; toxin)	
Day of infection	1st dol	3rd dol	1st dol	3rd dol
Litters 4 days post-infection				
	++ − +++ ^a^ [fimH; iucD (2 litters)]	++ - +++^a^ [fimH, papC, iucD, cnf1]	+ [A, beta 2]	+++ [A, beta 2]
Individual piglets (1st dol: *n* = 1+1, 3rd dol: *n* = 7)				
Diarrhoeic piglet(s), toltrazuril treated	(+) [fimH; iucD]	++ - +++^a^ [fimH, papC, iucD, cnf1]	Negative	+ − +++ [A, beta 2]
Matched healthy control	(+) [fimH]	Not done	Negative	Not done
Sows *ante partum*				
	++ − +++^a^ [fimH, papC, astA, iucD, cnf1]	Not done	Negative	Not done

^a^Haemolytic growth

*Abbreviations*: AF, autofluorescence; MM, McMaster; OpG, oocysts per gram of faeces; FS, faecal score; dol, day of life

## Discussion

In the present study, parenteral application of toltrazuril (in combination with gleptoferron for the prevention of iron-deficiency anaemia) on the 2nd dol was equally safe and effective compared to oral application on the 4th dol. Oocyst excretion was completely suppressed while the untreated control animals shed oocysts for one week on average. Diarrhoea (FS > 2) was reduced to a single animal in the Forceris® group; for this case no particular aetiology could be determined. In the Baycox® group, no animal had FS > 2. In contrast, all animals from the control group had diarrhoea for 3.6 days on average, signifying the clinical effect of coccidiosis. Due to a severe suppression of the body weight gain in the acute phase of infection in the untreated piglets, the overall body weight by the end of the study was reduced in this group, albeit not to a significant level.

The high efficacy of parenteral toltrazuril against porcine cystoisosporosis has previously been demonstrated in experimental and field trials [4, 16]. Since we wanted to compare the outcome of parenteral toltrazuril treatment on the 2nd dol against infection with *C. suis* on the 1st dol *versus* the 3rd dol, we employed the infection model using the same toltrazuril-sensitive *C. suis* strain and the same treatment regime as before [4]. Sows for both trials were purchased from the same breeder and had undergone the same vaccination scheme (erysipelas and porcine rotavirus vaccination, no immunization against *E. coli* or *Cl. perfringens*). Early infection on the 1st dol resulted in increased oocyst excretion in the control group both in terms of the duration and excretion intensity. This is in line with previous reports about the rapidly evolving age resistance against *C. suis* in piglets [11–13]. However, despite the strongly increased oocyst output, the parameters related to diarrhoea were comparable in both trials; for some even a slight increase in the animals infected later was seen. Apart from the infection time point, there was also a noticeable difference between the two trials with regard to bacterial co-infections. The quantity of *E. coli* and *Cl. perfringens* shed by the piglets four days after infection was much lower in the present study than in the previous one, where haemolytic *E. coli* expressing a set of four different virulence factors and beta 2 toxin-positive *Cl. perfringens* type A were abundant. After treatment, only low numbers of *E. coli* could be detected in the treated piglets with early infection, while in the previous study both *E. coli* and *Cl. perfringens* were abundant in treated diarrhoeic piglets.

Effective control of coccidial infections generally requires early intervention to limit tissue damage and environmental contamination with oocysts. Oral toltrazuril application in the first week of life to suckling piglets at risk of infection with *C. suis* shortly after birth can effectively prevent oocyst excretion and infection-related diarrhoea [1–4]. Metaphylactic treatment is usually conducted three to five days after birth as per product label [17]; however, in cases of high infection pressure with infections directly after birth it is advisable to treat as early as the 1st dol, since even in the prepatent period of parasite multiplication tissue damage can be considerable. This may be especially the case in the presence of enteropathogenic bacteria such as *Cl. perfringens* type A [14]. The mechanisms of interaction between *Cl. perfringens* and *C. suis* are not resolved; however, it appears that enteritis caused by coccidial infections promotes intestinal colonisation and (over)growth with *Cl. perfringens* and induces necrotic enteritis in piglets [14, 18] as well as in chickens [19]. Experimental studies in chickens have shown that infections with coccidia promote the growth of mucolytic bacteria, most probably due to the intestinal mucogenic response to parasite infection and leakage of glycoproteins and mannose residues from intestinal cells which promote the adhesion of pathogenic bacteria and together with other pro-inflammatory responses, result in necrotic enteritis (reviewed in [20]). An association of *Cl. perfringens* with eimeriosis in the pathogenesis of enteritis in cattle has also been described [21]. Interactions between *Cl. perfringens* and *C. suis* are supported by field observations [22] and therefore early treatment against *C. suis* on the day after birth is advisable in cases where neonatal infections are to be expected. Early toltrazuril treatment has also been suggested for prevention of coccidiosis necrotic enteritis in chickens [23].

## Conclusions

We assume that in the absence of *C. suis*, enteropathogenic bacteria may still cause some diarrhoea [18] and are the reason for “background” diarrhoeic piglets in toltrazuril-treated animals. From our data we also conclude that treatment of piglets infected with *C. suis* can reduce the pathogenic effects of the detected bacteria, as diarrhoea was highly and significantly reduced in toltrazuril-treated piglets in relation to the controls as shown in previous trials. Since the two different infection time points were investigated using different litters to avoid accidental cross-infections, no conclusions can be made as to whether this had any effect on the outcome of the parasite-bacteria interactions. Further studies on the interactions between *C. suis* and the gut microbiota in the suckling period are necessary to elucidate its underlying mechanisms in suckling piglets.

## Data Availability

Data supporting the conclusions of this article are included within the article. Raw data will not be shared as study documentation is protected by confidentiality agreements.

## Abbreviations

AF: autofluorescence
*C. suis*: *Cystoisospora suis*
FS: faecal score
OpG: oocysts per gram of faeces
SD: study day
